# Risk of Induction of Corneal Neovascularization with Topical Erythropoietin: An Animal Safety Study

**DOI:** 10.18502/jovr.v18i3.13772

**Published:** 2023-07-28

**Authors:** Sepehr Feizi, Mozhgan Rezaei Kanavi, Mohammad Abolhosseini, Seyed-Mohamadmehdi Moshtaghion, Hamed Esfandiari

**Affiliations:** ^1^Ocular Tissue Engineering Research Center, Research Institute for Ophthalmology and Vision Science, Shahid Beheshti University of Medical Sciences, Tehran, Iran; ^2^Ophthalmic Research Center, Research Institute for Ophthalmology and Vision Science, Shahid Beheshti University of Medical Sciences, Tehran, Iran; ^3^Department of Ophthalmology, Olmsted Medical Center, Rochester, Minnesota, USA

**Keywords:** Chemical Burns, Corneal Neovascularization, Rabbit Cornea, Topical Erythropoietin

## Abstract

**Purpose:**

To evaluate the pro-angiogenic effect of topical erythropoietin on cornea in chemical burn-injured rabbit eyes.

**Methods:**

The corneal alkali-burn injury was induced in 10 eyes of 10 rabbits using filter paper saturated with 1.0 mol sodium hydroxide. The eyes were categorized into the treatment group (*n* = 5) that received topical erythropoietin (3000 IU/mL) every 8 hr for one month versus the control group (*n* = 5) that received normal saline every 8 hr for one month. All eyes were treated with topical ciprofloxacin every 8 hr until corneal re-epithelialization was complete. Corneal epithelial defects, stromal opacity, and neovascularization were evaluated after the injury. At the conclusion of the study, the rabbits were euthanized and their corneas were submitted to histopathological examination.

**Results:**

Baseline characteristics including the rabbits' weight and the severity of corneal injury were comparable in two groups. Time to complete corneal re-epithelialization was 37 days in the treatment group and 45 days in the control group (*P* = 0.83). There was no significant difference between the groups in the rate of epithelial healing or corneal opacification. Clinical and microscopic corneal neovascularization was observed in one eye (20%) in the treatment group and two eyes (40%) in the control group (*P* = 0.49).

**Conclusion:**

Recombinant human erythropoietin administered topically did not induce vessel formation in rabbit corneas after chemical burn.

##  INTRODUCTION

Erythropoietin is a glycoprotein hormone that regulates erythropoiesis by stimulating the differentiation and proliferation of hematopoietic stem cells and inhibiting their apoptosis.^[1]^ Production of this hormone is not limited to the hematopoietic tissues. Other non-hematopoietic tissues such as vascular endothelium and retina can produce erythropoietin or express its specific receptor in response to hypoxia and biochemical and physical stress indicating the role of this hormone in physiological homeostasis.^[2,3,4,5,6]^ Erythropoietin has multiple biological functions including its ability to reduce excitotoxicity, oxidation, inflammation, and apoptosis as well as upregulating the proliferation of progenitor cells.^[7]^

This glycoprotein hormone has been used in the treatment of different ocular diseases such as optic neuritis, methanol optic neuropathy, glaucoma, retinopathy of prematurity (ROP), retinal detachment, diabetic retinopathy, traumatic optic neuropathy, central and branch retinal vein occlusion, and non-arteritic anterior ischemic optic neuropathy.^[7]^ Recently, we reported the efficacy of topical erythropoietin for the management of scleral necrosis caused by different etiologies.^[8,9,10]^ The results of our experimental study showed that topical erythropoietin induced vascularization of the necrotic sclera and reduced the inflammatory response and apoptosis.^[8]^ One concern of using topical erythropoietin in the management of scleral necrosis is corneal neovascularization. Several studies have reported retinal neovascularization in eyes with severe proliferative diabetic retinopathy (PDR) and ROP after treatment with erythropietin.^[7]^ These reports can suggest a potential association with pathological neovascularization in other ocular tissues such as the cornea. Abnormal expression of erythropoietin in the cornea is shown to contribute to the corneal neovascularization in animal models suggesting a role for erythropoietin-antagonizing agents in the treatment of this complication.^[11,12]^ Other animal and human studies, however, did not replicate these results.^[8,9,10][13]^ Given the conflicting data regarding the pro-angiogenic effect of this hormone on the cornea, we set this experiment to investigate the effect of topical erythropoietin on corneal neovascularization in rabbit eyes.

##  METHODS

Ten healthy male New Zealand albino rabbits, 12–18 months of age, and weighing 1–3 kg were included in this study. The rabbits were kept under standard conditions (24 ± 2ºC, 12 hr light/12 hr dark cycles, 55 ± 10% humidity) and received human care as outlined in the ARVO Statement for the Use of Animals in Ophthalmic and Vision Research. The study was approved by the Ophthalmic Research Center, affiliated with the Shahid Beheshti University of Medical Sciences in Tehran, Iran. The animals were adapted for one week prior to the experiment, and a pellet diet (standard rodent diet) and water were provided ad libitum.

### Induction of Chemical Burns

Twelve hours after food withdrawal, the rabbits were anesthetized with an intramuscular injection of a mixture of 3.5 mg/kg ketamine (Ketamine 10%Ⓡ, Kepro, Deventer, The Netherlands), 5 mg/kg xylazine (Xylazine 2%Ⓡ, Alfasan, The Netherlands), and 1 mg/kg acepromazine (Neurotrang 1%Ⓡ, Alfasan, The Netherlands). Eyes were topically anesthetized with 1% tetracaine hydrochloride ophthalmic solution. To induce corneal chemical burns, a round filter paper disc (8 mm in diameter) that was immersed in 1 mol sodium hydroxide for 10 s was placed on the cornea for 60 s. The cornea and the conjunctival sac were then irrigated copiously with 80 mL of phosphate-buffered saline solution for at least 5 min until pH, measured with pH paper, decreased to 7.0–7.5 range.

**Figure 1:**
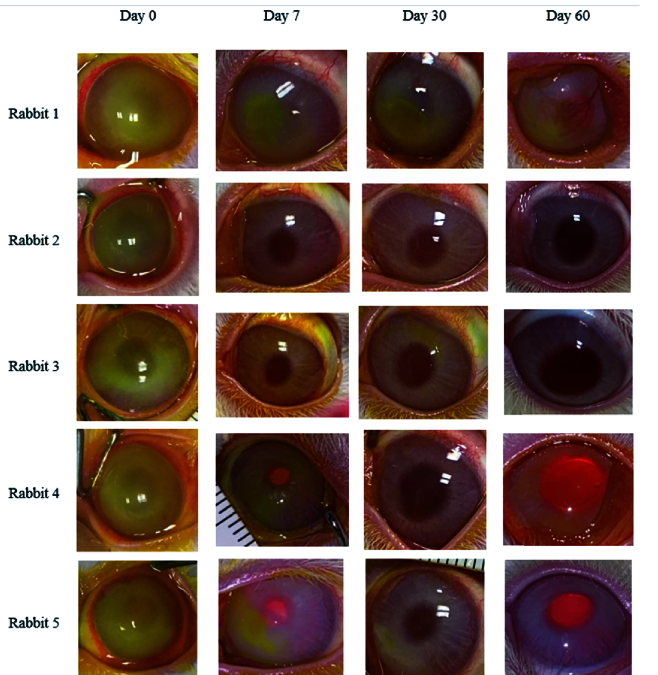
Diffuse photographs demonstrate changes in the size of corneal epithelial defects and stromal opacity in the treatment group. Only one eye (rabbit 1) had epithelial defects and stromal opacity and neovascularization at the final examination.

**Figure 2:**
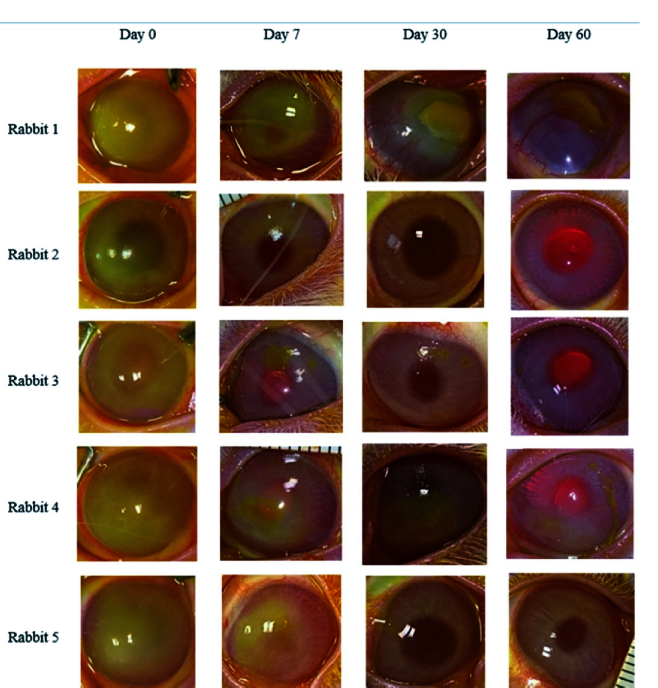
Diffuse photographs demonstrate changes in the size of corneal epithelial defects and stromal opacity in the control group. Two eyes (rabbits 1 and 4) had epithelial defects and stromal opacity and neovascularization at the final examination.

**Figure 3:**
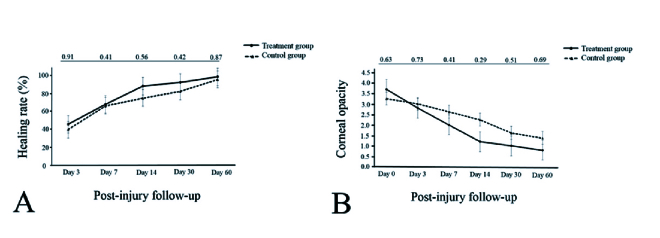
Comparison of healing rate of corneal epithelial defects (A) and stromal opacity (B) between the treatment and control groups. *P-*values for the comparison between two groups are provided at the top of the figures.

**Figure 4:**
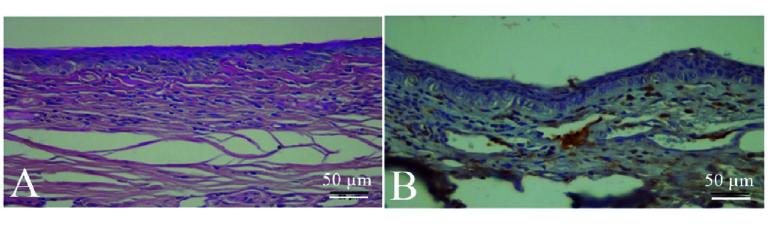
Histological illustrations of the rabbit cornea with stromal neovascularization. (A) Hematoxylin and eosin staining demonstrates a minute migration of the epithelium at the edge of the wound, stromal edema with mild polymorphonuclear leukocyte infiltration, and new blood vessel formation. (B) Immunohistochemical examination reveals CD31 immune reactivity of the peripheral corneal stroma.

**Table 1:** Comparison of the study groups in the area of corneal epithelial defects measured at different time points after alkali injury.


**Examination day**	**Area of corneal epithelial defects (mm^2^)**		***P*** **-value**
	**Treated group**	**Control group**	
0	135.1 ± 20.3	106.9 ± 21.4	0.11
3	73.0 ± 13.4	61.9 ± 16.1	0.41
7	43.3 ± 14.2	33.3 ± 28.7	0.29
14	15.4 ± 20.2	24.1 ± 31.8	0.56
30	10.2 ± 20.6	16.7 ± 20.7	0.73
60	2.1 ± 4.7	4.1 ± 5.2	0.47
	
	
mm^2^, square millimeters			

### Post-injury Examination and Treatment Measures

Immediately after corneal chemical burn induction, the rabbits were randomly divided into one of the two groups; the treatment group (*n* = 5) that received erythropoietin eye drops every 8 hr for one month and the control group (*n* = 5). Isotonic normal saline was administered in the control group every 8 hr for one month. All rabbits received topical ciprofloxacin 0.3% every 8 hr until corneal re-epithelialization was complete to prevent bacterial keratitis. Erythropoietin eye drops were prepared under sterile conditions by diluting 1.5 mL of commercially available recombinant human erythropoietin solution (PDpoetin, 10,000 IU/0.5 mL; Pooyesh Darou Biopharmaceutical Co., Tehran, Iran) with 8.5 mL of isotonic normal saline to achieve a final concentration of 3000 IU/mL. Topical erythropoietin drops were freshly reconstituted every three days and kept in a refrigerator (4–8ºC).

Post-injury examinations were performed at days 0, 3, 7, 14, 30, and 60 after the chemical burn using a surgical microscope. We used 1% fluorescein sodium solution and cobalt blue light to stain and delineate the border of corneal epithelial defects. The photographs obtained at each follow-up examination were evaluated for corneal epithelial defects, severity of corneal stromal opacity, and presence of corneal neovascularization. The ImageJ software (National Institutes of Health, Bethesda, Md., USA) was used to calculate the area of corneal epithelial defects at each examination. The rate of corneal epithelial healing was calculated using the following formula:

Corneal re-epithelialization rate = [(A0 – A) / A0] × 100, where A0 is the area of epithelial defects measured at 0 hr after chemical burn and A is the area of epithelial defects measured at each follow-up visit. Corneal opacity was evaluated and scored based on a scale proposed by Holland et al.^[14]^ The scoring system for clarity was 0, clear cornea; 1, very slight opacity; 2, slight opacity with visible iris vessels and pupil; 3, moderate opacity with only the visibility of the pupil; and 4, heavy opacity with no visibility of iris and crystalline lens.

### Histopathological Examination of the Cornea

The rabbits were euthanized after an intracardiac injection of 1 mL (60 mg) pentobarbital sodium on the postoperative day 60. The eyes were enucleated immediately and fixed in 10% neutral buffered formalin for 24 hr. Then, the corneoscleral disc was removed, bisected into two halves, processed, and embedded in a paraffin block. Several 5 µm-tissue sections at three different levels (250 µm apart) were prepared and stained with hematoxylin-eosin and periodic acid Schiff and evaluated under a light microscope (BX41, Olympus, Tokyo, Japan) which was equipped with a digital camera (DP12 Microscope Camera, Olympus, Tokyo, Japan). The histopathological features that were examined included corneal epithelial healing, the presence of goblet cells in the corneal epithelium, the presence of inflammatory cells, and the severity of corneal stromal vascularization. Immunohistochemical staining for CD31 was performed to identify the presence of blood vessels, as previously described.^[8]^ A fluorescence microscope (Olympus IX71, Tokyo, Japan) with an excitation wavelength ranging from 450 to 490 nm was used to evaluate the CD31-stained sections.

### Statistical Analysis

Statistical analyses were performed using SPSS statistical software version 24 (IBM Corp., Armonk, NY, USA). The Mann–Whitney test was used to compare the rabbits' weight measured at the beginning of the study as well as the area of the epithelial defects measured at each follow-up. The mean time to complete corneal epithelialization was compared between the groups using a Kaplan–Meier survival curve and log-rank test. *P* < 0.05 was considered the threshold for statistical significance.

##  RESULTS

The mean rabbit weight was 1870 ± 234 gr in the treated group and 1917 ± 289 gr in the control group (*P* = 0.56). All eyes had clinical signs of alkali burn including conjunctival injection, corneal epithelial defects, and grayish, white, cloudy stromal haziness immediately after the injury [Figures 1 & 2]. Both groups were comparable in the area of corneal epithelial defects measured immediately after chemical burn and at different time points [Table 1]. The rate of corneal re-epithelialization was 45.6% ± 1.5%, 67.7% ± 10.7%, 88.1% ± 15.7%, 92.1% ± 15.9%, and 98.4% ± 0.04% at 3, 7, 14, 30, and 60 days after injury in the erythropoietin-treated group, respectively [Figure 3A]. The corresponding numbers were 39.7% ± 21.9%, 66.3% ± 31.2%, 74.8% ± 33.5%, 82.1% ± 21.9%, and 95.5% ± 0.1% in the control group, with no statistically significant difference between the two groups at any time point. Corneal epithelial defects completely improved in four eyes (80%) in the treated group and three eyes (60%) in the control group during the course of the study (*P* = 0.51). The Kaplan–Meier survival curve was used to calculate the mean interval until complete healing of corneal epithelial defects, which was 37 days in the treated group and 45 days in the control group (*P* = 0.83).

Figure 3B demonstrates and compares the degree of corneal opacity between the groups. There was no significant difference in the degree of corneal opacity throughout the study. At the final examination, corneal opacity was completely resolved in three eyes (60%) in each group (*P* = 1.0).

Clinical corneal neovascularization developed in one eye (20%) in the treatment group and two eyes (40%) in the control group (*P* = 0.49) [Figures 1 & 2]. All of these three eyes had non-healing epithelial defects at the final examination. Corneal neovascularization was first observed on day 30 in the eye in the treatment group; these vessels involved 4 clock hr of the stroma and advanced to the central cornea at the final follow-up examination. Corneal neovascularization was first noted on day 14 in the two control eyes. At the final follow-up examination, neovascularization involved 3 clock hr of the corneal periphery in one eye and of the total cornea in the other eye of the control group. No other complications such as corneal infection or perforation were observed in any group.

### Histological Evaluation of the Corneas

Light microscopic examinations of seven corneas with complete healing of epithelial defects demonstrated intact and morphologically normal epithelium with 3–4 layers of flat and uniformly arranged epithelial cells. There was no obvious angiogenesis in these corneas, however, there were a few infiltrations of polymorphonuclear leukocytes in the corneal stroma. Descemet's membrane and the endothelium did not show any structural changes.

The corneal epithelium was absent in some areas in one eye of the treated group and two eyes of the control group. Corneal stroma of these three eyes demonstrated considerable keratitis as evidenced by the infiltration of inflammatory cells and loss of keratocytes. In addition, proliferating blood vessels surrounded by inflammatory cells were observed in the anterior corneal stroma. We also evaluated the expression of the CD31 antigen to identify neovascularization, which confirmed the presence of blood vessels in corneal stroma in these three eyes [Figure 4]. The other corneas without vascularization on gross and microscopic examinations failed to demonstrate CD31 immunoreactivity, indicating the lack of subclinical corneal angiogenesis in these eyes. Goblet cells were not observed in the corneal epithelium of any study eyes, indicating the lack of limbal stem cell deficiency.

##  DISCUSSION

Corneal neovascularization is a serious complication that can interfere with normal vision and reduce the success of corneal allografts.^[15,16,17]^ The regulation of corneal angiogenesis is a complex process which involves a delicate balance between pro- and anti-angiogenic agents.^[18]^ A pro-angiogenic shift can occur due to various corneal insults such as trauma, chemical burn, and infection. Several angiogenic factors including basic fibroblast growth factor, vascular endothelial growth factor, and transforming growth factor-α and -β are shown to enhance corneal neovascularization.^[18]^ Recently, the role of erythropoietin in ocular abnormal angiogenesis has attracted considerable attention. It has been shown that the activation of the erythropoietin receptors facilitates proliferation, differentiation, and mobilization of resident endothelial cells.^[2,4,6,19]^ Furthermore, this hormone prevents apoptosis of the endothelial cells, reestablishes their intercellular tight junction proteins, and regulates the traffic of endothelial progenitor cells to the injury site.^[2,6,20]^ Elevated levels of erythropoietin were found in the vitreous samples of patients with PDR and ROP.^[20]^ The literature review regarding the effects of erythropoietin on the corneal neovascularization is inconclusive. Some experimental studies have illustrated pro-angiogenic effects of erythropoietin on the cornea and suggested the use of erythropoietin-antagonizing agents in the treatment of corneal neovascularization. Livny et al^[11]^ applied topical erythropoietin every 8 hr for ≤ 4 days in a rabbit model of corneal epithelial defects. While clinical corneal neovascularization was not observed after treatment, histological evaluation demonstrated neovascularization of the anterior stroma in two of the six erythropoietin-treated eyes as compared to none in the control eyes.^[11]^ In their experiment on a murine model, Luo et al^[12]^ found high levels of erythropoietin and its receptor in vascularized corneas after chemical burn. In addition, they found moderate corneal angiogenesis after intrastromal injection of erythropoietin.^[12]^ These results were not replicated in other animal and human studies. Sinclair et al^[13]^ found that clinical neovascularization was not induced by nylon discs coated with mouse recombinant erythropoietin that were embedded in the rat corneas. However, no histological assessment was performed in that study.^[13]^ In our recent experimental study, we applied topical erythropoietin every 8 hr for 30 days in a rabbit model of surgically induced necrotizing scleritis.^[8]^ We observed no gross or microscopic corneal neovascularization.^[8]^ In our human studies, we used topical erythropoietin four times a day to treat scleral necrosis caused by different etiologies.^[9,10]^ Our studies suggested that topical erythropoietin did not result in corneal neovascularization when administered in intact corneal epithelium.^[9,10]^ Corneal epithelial defect and limbal stem cell deficiency, however, were both associated with corneal neovascularization after application of topical erythropoietin.^[10]^

In the current study, we investigated the corneal safety of topical erythropoietin in an alkali injury model *in vivo*. The protocol we used to induce chemical burn (1 mol sodium hydroxide for 60 s) was previously used in several studies.^[21,22]^ We found that topical erythropoietin did not hasten corneal epithelial defect healing nor reduce stromal opacity in rabbit eyes following chemical burn. These results are consistent with Livny et al's study.^[11]^ In the current study, we found that treatment with topical erythropoietin did not increase the rate of clinical and histological corneal angiogenesis. Corneal neovascularization developed in one eye in the treatment group and two eyes in the control group. These eyes had persistent epithelial defects and inflammation, indicating that neovascularization may have been the result of a severe inflammatory response. The lack of an angiogenesis effect of topical erythropoietin in our animal study could be due to unresponsive rabbit receptors to recombinant human erythropoietin. However, in our previous study, we showed recombinant human erythropoietin applied at the same concentration and frequency could effectively induce conjunctival and scleral vascularization in rabbit eyes.^[8]^ In addition, one can argue that higher doses and frequency of drug application could have elicited the angiogenesis response.

Our finding that topical erythropoietin did not lead to corneal neovascularization can also be explained by the anti-angiogenic effects of normal limbal stem cells.^[23,24,25]^ Duan et al^[23]^ demonstrated that an intact limbus of rabbit eyes contains high levels of anti-angiogenic agents that can prevent corneal angiogenesis. Similarly, it has been demonstrated that normal human limbus secretes multiple anti-angiogenic factors and has many complex extracellular matrix–cell and cell–cell interactions that maintains a corneal avascular state.^[24,25]^ This is in line with our recent clinical experience demonstrating that topical erythropoietin promoted corneal neovascularization only in human eyes with limbal stem cell deficiency caused by thermal or chemical burns.^[10]^

In summary, there was no significant difference between the study groups in terms of corneal neovascularization, indicating that topical erythropoietin 3000 U/mL applied three times a day for 30 days did not promote angiogenesis in the alkali-injured rabbit corneas. The limitation of this study is that the sample size is too small. Moreover, we used only one dose of the medication. Induction of corneal neovascularization can be a dose-dependent response and may develop with a higher concentration and more frequent application of erythropoietin. Further studies are warranted to investigate whether higher concentrations and more frequent applications of topical erythropoietin could result in corneal neovascularization.

### Ethical Considerations

All procedures performed in the study involving animal participants were done in accordance with the ARVO Statement for the Use of Animals in Ophthalmic and Vision Research. The study was approved by the Ophthalmic Research Center, affiliated with the Shahid Beheshti University of Medical Sciences in Tehran, Iran.

##  Financial Support and Sponsorship

The authors received no financial support for the research, authorship, and/or publication of this article.

##  Conflicts of Interest 

The authors declared no potential conflicts of interest with respect to the research, authorship, and/or publication of this article.
